# Effective prime factorization via quantum annealing by modular locally-structured embedding

**DOI:** 10.1038/s41598-024-53708-7

**Published:** 2024-02-12

**Authors:** Jingwen Ding, Giuseppe Spallitta, Roberto Sebastiani

**Affiliations:** Department of Computer Science and Engineering, University of Trento, Trento, Italy

**Keywords:** Computer science, Computational science

## Abstract

This paper investigates novel techniques to solve prime factorization by quantum annealing (QA). First, we present a very-compact modular *encoding* of a multiplier circuit into the architecture of current D-Wave QA devices. The key contribution is a compact encoding of a controlled full-adder into an 8-qubit module in the Pegasus topology, which we synthesized using Optimization Modulo Theories. This allows us to encode up to a 21 × 12-bit multiplier (and a 22 × 8-bit one) into the Pegasus 5760-qubit topology of current annealers. To the best of our knowledge, these are the largest factorization problems ever encoded into a quantum annealer. Second, we investigated the problem of actually *solving* encoded PF problems by running an extensive experimental evaluation on a D-Wave Advantage 4.1 quantum annealer. In the experiments we introduced different approaches to initialize the multiplier qubits and adopted several performance enhancement techniques. Overall, 8,219,999 = 32,749 × 251 was the highest prime product we were able to factorize within the limits of our QPU resources. To the best of our knowledge, this is the largest number which was ever factorized by means of a quantum annealer; also, this is the largest number which was ever factorized by means of any quantum device without relying on external search or preprocessing procedures run on classical computers.

## Introduction

### Motivations

*Integer factorization (IF)* is the problem of factoring a positive integer into a product of small integers, called factors. If the factors are restricted to be prime, we refer to it as *prime factorization (PF)*. Finding prime factors becomes increasingly difficult as the numbers get larger. In particular, the state-of-the-art classical algorithm to solve PF is the *general number field sieve* algorithm^1^, which has sub-exponential time complexity^2^. Even though PF is not believed to be NP-complete, no polynomial-time classical algorithm solving it has been presented in the literature. The hardness of prime factorization is exploited in modern cryptography, where it is used as a basis for secure encryption algorithms (e.g. the RSA public-key encryption^3^) since the process of factoring large numbers is currently considered computationally infeasible for classical computers.

### State of the art

*Quantum computers* have the potential to perform PF exponentially faster than classical computers. A first approach in tackling PF by quantum computing is *Shor’s algorithm*^4^. This technique takes advantage of the properties of quantum mechanics, such as superposition and entanglement, to factor numbers into their prime factors in poly-logarithmic time. Although several efforts in implementing this algorithm, and variations thereof, on existing gate-based quantum computers have been presented in the literature^5–9^, the size of IP/PF which were actually implemented and solved on quantum devices is very small, in the order of a few thousand. Notice that a large-scale simulation of Shor’s algorithm on a GPU-based classical supercomputer allowed factorization up to 549,755,813,701^10^.

Another approach consists in relying on *variational methods*, a form of hybrid classical-quantum procedure. Variational algorithms use parameterized quantum circuits, where the gates in the circuit are associated with adjustable parameters. These parameters act as variables of a certain cost function, which quantifies the difference between the desired quantum state (the ground state) and the state produced by the parameterized circuit. The goal is to adjust the parameters and minimize this cost function through an optimization process.

A couple of papers solved prime factorization on top of variational approaches: a first attempt allowed for the factorization of 91 in the IBMQ hardware^11^; with the integration of an aggressive pre-processing phase, which is performed by classical computation, the factorization of the three following biprime numbers were achieved: 3127 (53 $\times$ 59), 6557 (79 $\times$ 83), and 1,099,551,473,989 (1,048,589 $\times$ 1,048,601)^12^. These numbers, however, have some peculiar characteristics that can make their factorization easy^11,13^: they can be easily factorized through the Fermat factorization technique^14^, so that an aggressive pre-processing phase might heavily reduce the size of the problem to be embedded in the quantum circuit.

*Quantum Annealing (QA)*^15^ has shown to be effective in performing prime factorization, e.g., by reducing high-degree cost functions to quadratic either by using Groebner bases^16^ or by using equivalent quadratic models produced by adding ancillary variables^17^, or by related approaches^18^. Currently, the largest factorization problem mapped to the quantum annealer D-Wave 2000Q is 376,289. Moreover, all bi-primes up to 200,000 have been solved by D-Wave 2X processors^16,17^. Also, by using D-Wave *hybrid Classical-QA* tool, 1,005,973 has been factored^19^.

We refer the reader to Willsch et al.^10^ for a recent very detailed survey on solving PF with quantum devices.

### Contributions

In this paper, we propose a novel approach based on a modular version of locally-structured embedding of satisfiability problems^20,21^ to encode IF/PF problems into Ising models and solve them using QA. Our contribution is twofold.

First, we present a novel modular *encoding* of a binary multiplier circuit into the architecture of the most recent D-Wave QA devices. The key contribution is a compact encoding of a controlled full-adder into an 8-qubit module in the Pegasus topology^22^, which we synthesized offline by means of Optimization Modulo Theories. The multiplier circuit is then built by exploiting a bunch of novel ideas, namely *alternating modules*, *qubit sharing* between neighboring modules, and *virtual chaining* between non-coupled qubits. This allows us to encode up to a 21 $\times$ 12-bit multiplier (resp. a 22 $\times$ 8-bit one) into the Pegasus 5760-qubit topology of current annealers, so that a faulty-free annealer could be fed an integer factorization problem up to 8,587,833,345 = 2,097,151 $\times$ 4095 (resp. 1,069,547,265 = 4,194,303 $\times$ 255)), allowing for prime factorization of up to 8,583,606,299 = 2,097,143 $\times$ 4093 (resp. 1,052,769,551 = 4,194,301 $\times$ 251). To the best of our knowledge, these are the largest factorization problems ever encoded into a quantum annealer. We stress the fact that, given the modularity of the encoding, this number will scale up automatically with the growth of the qubit number in future chips.

Second, we have investigated the problem of actually *solving* encoded PF problems by running an extensive experimental evaluation on a D-Wave Advantage 4.1 quantum annealer. Due to faulty qubits and qubit couplings of the QA hardware we had access to, it was possible to feed to it at most a 17 $\times$ 8-bit multiplier, corresponding to at most a 33,423,105 = 131,071 $\times$ 255 factorization. To help the annealer in reaching the global minimum, in the experiments we introduced different approaches to initialize the multiplier qubits and adopted several performance enhancement techniques, like *thermal relaxation*, *pausing*, and *reverse annealing*, which we combined together by iterative strategies, discussing their synergy when combined. Overall, exploiting all the encoding and solving techniques described in this paper, 8,219,999 = 32,749 $\times$ 251 was the highest prime product we were able to factorize within the limits of our QPU resources. To the best of our knowledge, this is the largest number which was ever factorized by means of a quantum annealer; also, this is the largest number which was ever factorized by means of any quantum device

without relying on external search or preprocessing procedures run on classical computers.

*Disclaimer.* Due to space constraints, some details in some figures may not be easy to grasp from a printed version of this paper. Nevertheless, all figures are high-resolution ones, so that every detail can be grasped in full if they are seen via a pdf viewer.

## Foundations

### D-wave quantum annealers

From a physicist’s perspective, D-Wave’s quantum annealers (QAs) are quantum devices that use quantum phenomena to reach minimum-energy states in terms of the values of their *qubits* (i.e. minimum-energy states of superconducting loops).

For these QAs, the (quantum) *Hamiltonian* *H*(*s*) —which corresponds to the classical Hamiltonian that described some physical system in terms of its energies—is represented by the sum of the driver Hamiltonian $H_{driver}$ and the classical Ising Hamiltonian $H_{Ising}$, where $\hat{\sigma }_{x,z}^{(i)}$ are Pauli matrices operating on a qubit $q_i$, such that $h_i$ and $J_{i, j}$ are programmable parameters representing the qubit biases and coupling strengths:

1 $$\begin{aligned} \textstyle H(s) {\mathop {=}\limits ^{\text {\tiny def}}}-\frac{A(s)}{2} H_{driver} + \frac{B(s)}{2} H_{Ising},\ \ \ \ {such\ that\ }\ H_{driver} {\mathop {=}\limits ^{\text {\tiny def}}} \sum _i \hat{\sigma }_x^{(i)}, \ H_{Ising} {\mathop {=}\limits ^{\text {\tiny def}}} \sum _i h_i \hat{\sigma }_z^{(i)} + \sum _{i>j} J_{ij} \hat{\sigma }_z^{(i)} \hat{\sigma }_z^{(j)}. \end{aligned}$$

**Figure 1:**
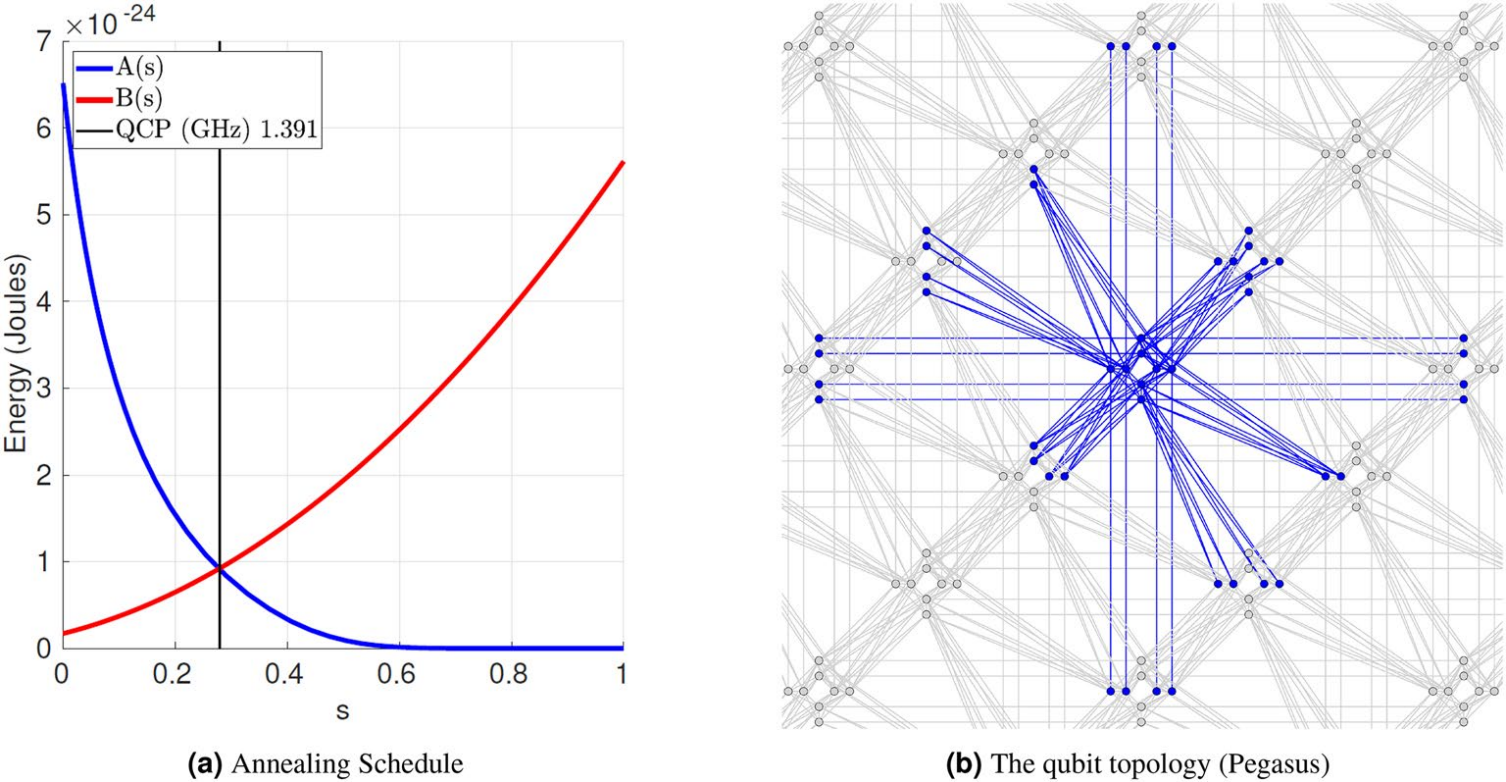
Information about the D-Wave Pegasus systems. In Fig. 1a, *s* stands for the normalized anneal fraction time..

The parameter *s* is the normalized anneal fraction, $s=t/t_f \in [0, 1]$, where *t* is time and $t_f$ is the total time of the annealing process. This *s*-dependent Hamiltonian *H*(*s*) smoothly interpolates between $H_{driver}$ and $H_{Ising}$ through the two annealing functions *A*(*s*), *B*(*s*), as shown in Fig. 1a. At $s=0$, the system starts in the ground state of $H_{driver}$, with all qubits in the superposition state of 0 and 1; as the system is annealed $s \uparrow$, the dominance of $H_{driver}$ decreases and $H_{Ising}$ comes to play; at the end of the annealing process $s=1$, the system would end up in a classical state that corresponds to $H_{Ising}$. According to the quantum adiabatic theorem, the system will remain in the instantaneous groundstate through the evolution iff the system is annealed slowly enough. The required runtime according to the theorem is proportional to $\frac{1}{gap^2}$, where *gap* is the minimal gap between the ground state and excited states during the system’s evolution.

From a computer scientist’s perspective, D-Wave’s QAs are specialized quantum computers which draws optima or near-optima from quadratic cost functions on binary variables, that is, specialized hardware for solving the *Ising problem*^21^:

2 $$\begin{aligned} \textstyle argmin_{\underline{\textbf{z}} \in \{{-1,1}\} ^{|V|}}\ H(\underline{\textbf{z}}),{} & {} \textstyle {such\ that\ } \ H(\underline{\textbf{z}}) {\mathop {=}\limits ^{\text {\tiny def}}} \sum _{i \in V} h_i z_i + \sum _{\begin{array}{c} (i,j)\in E \end{array}} J_{i,j} z_i z_j, \end{aligned}$$

where each variable $$z_i\in \{{-1,1}\} $$ is associated with a qubit; $$G=\langle {V,E}\rangle $$ is an undirected graph, *the hardware graph* or *topology*, whose edges correspond to the physically-allowed qubit interactions; and $$ h_i$$, $$J_{i,j}$$ are programmable real-valued parameters.

The current Pegasus topology^22^ was introduced in the D-Wave Advantage quantum annealing machine and is based on a lattice of qubits. The lattice is divided into cells (“tiles”), where each cell contains eight qubits arranged in a bipartite graph. We call qubits on the same side of the partition either *vertical* or *horizontal* qubits. Qubits of the same side inside each tile are connected 2-by-2. Figure 1b shows the Pegasus topology for a 3 $\times$ 3 subgraph. It extends the previous Chimera topology by adding more connections between the tiles so that the degree of connectivity of each qubit is up to 15. In particular, each tile is now connected to diagonally neighboring tiles through $45^\circ$, $120^\circ$ and $150^\circ$ connections among qubits w.r.t. the *x* axis (we will refer to them as *diagonal couplings*). Moreover, the configurable range of coefficients also increases, e.g., D-Wave Advantage 4.1 systems allow for biases and couplings such that $h_i\in [-4, 4]$ and $J_{i,j}\in [-2, 1]$.

### Monolithic encoding of small SAT problems based on OMT

Bian et al.^21^ formulated the problem of encoding SAT problems into *Ising models* that are compatible with the available quantum topology —represented as a graph (*V*, *E*) such that the nodes *V* are the qubits and the edges *E* are the qubit couplings— with the goal of feeding them to the quantum annealer. Here we briefly summarize their techniques, adopting the same notation.

Given a (small enough) Boolean formula $F(\underline{\textbf{x}})$ and a set of extra Boolean variables $\underline{\textbf{a}}$ (called *ancillae*), we first need to map the Boolean variables $\underline{\textbf{x}}$ and $\underline{\textbf{a}}$ into a subset $\underline{\textbf{z}} \subseteq V$ of the qubits in the topology, with the intended meaning that the qubit values $\{{1,-1}\}$ are interpreted as the truth values $\{{\top ,\bot }\}$ respectively. (With a little abuse of notation, we consider this map implicit and say that $\underline{\textbf{z}} {\mathop {=}\limits ^{\text {\tiny def}}} \underline{\textbf{x}} \cup \underline{\textbf{a}}$.) This map, called *placement*, can be performed either manually or via ad-hoc procedures^21^.

Then we need to compute the values $\theta _0$, $\theta _i$, and $\theta _{ij}$ of a *penalty function* $P_F(\underline{\textbf{x}},\underline{\textbf{a}} |\underline{\varvec{\theta }})$ such that, for some value $g_{min}>0$:

3 $$\begin{aligned} \textstyle P_F(\underbrace{\underline{\textbf{x}},\underline{\textbf{a}}}_{\underline{\textbf{z}}}|\underline{\varvec{\theta }}) {\mathop {=}\limits ^{\text {\tiny def}}} \theta _{0}{} + \sum _{\begin{array}{c} z_i\in V \end{array}} \theta _{i} z_i + \sum _{\begin{array}{c} (z_i,z_j)\in E, i<j \end{array}} \theta _{ij} z_i z_j; \ z_i \in \{-1, 1\};{} & {} \forall \underline{\textbf{x}}\ \ min_{\{{\underline{\textbf{a}}}\}} P_F(\underline{\textbf{x}},\underline{\textbf{a}} |\underline{\varvec{\theta }}) {\left\{ \begin{array}{ll} = 0 &{}\text { if } F(\underline{\textbf{x}})=\top \\ \ge g_{min} &{}\text { if } F(\underline{\textbf{x}})=\bot \end{array}\right. } \end{aligned}$$

Intuitively, $P_F(\underline{\textbf{x}},\underline{\textbf{a}} |\underline{\varvec{\theta }})$ allows for discriminating truth values for $\underline{\textbf{x}}$ which satisfy the original formula $F(\underline{\textbf{x}})$ (i.e., these such that $min_{\{{\underline{\textbf{a}}}\}} P_F(\underline{\textbf{x}},\underline{\textbf{a}} |\underline{\varvec{\theta }}) =0$) from these who do not (i.e., these such that $min_{\{{\underline{\textbf{a}}}\}} P_F(\underline{\textbf{x}},\underline{\textbf{a}} |\underline{\varvec{\theta }}) \ge g_{min}$). $\theta _0$, $\theta _i$, $\theta _{ij}$ and $g_{min}$ are called respectively *offset*, *biases*, *couplings* and the *gap*; the offset has no bounds, whereas biases and couplings have a fixed range of possible values ($[-2,+2]$ for biases and $[-1,+1]$ for coupling for the old Chimera architecture, $[-4,+4]$ for biases and $[-2,+1]$ for couplings for the Pegasus architecture of Advantage systems).

The penalty function $P_F(\underline{\textbf{x}},\underline{\textbf{a}} |\underline{\varvec{\theta }})$ (3) is fed to the quantum annealer, which tries to find values for the $\underline{\textbf{z}}$ ’s which minimizes it. Once the annealer reaches a final configuration, if the corresponding energy is zero, then we can conclude that the original formula is satisfiable and the values of $\underline{\textbf{x}} \subseteq \underline{\textbf{z}}$ satisfy $F(\underline{\textbf{x}})$—once reconverted from $\{{1,-1}\}$ to $\{{\top ,\bot }\}$. Notice that we may have a solution for $F(\underline{\textbf{x}})$ even if the energy of the assignment is not zero, because the truth values of the ancillae do not impact the satisfiability of the original formula $F(\underline{\textbf{x}})$ but may affect the final energy. (We will call them “$>0$-energy solutions”.) This is not an issue, because checking if the truth assignments of the variables in $\underline{\textbf{x}}$ satisfy $F(\underline{\textbf{x}})$ is trivial. Notice also that, since the annealer is not guaranteed to find a minimum, if the result is not a solution, then we cannot conclude that $F(\underline{\textbf{x}})$ is unsatisfiable.

The gap $g_{min}$ between ground and non-ground states has a fundamental role in making the annealing process more effective: the bigger $g_{min}$, the easier is for the annealer to discriminate between satisfying and non-satisfying assignments. Ancillae $\underline{\textbf{a}}$ are needed to increase the number of $\theta$ parameters, because the problem of finding a suitable $P_F(\underline{\textbf{x}},\underline{\textbf{a}} |\underline{\varvec{\theta }})$ matching (3) is over-constrained in general, so that without ancillae there would be no penalty function even for very few variables $\underline{\textbf{x}}$ ’s (e.g., $>3$). The more ancillae, the more degrees of freedom, the higher the chances to have a suitable penalty with a higher gap $g_{min}$.

The problem of synthesizing $P_F(\underline{\textbf{x}},\underline{\textbf{a}} |\underline{\varvec{\theta }})$ is solved by using a solver for *Optimization Modulo Theories* such as OptiMathSAT^23^. For the Pegasus architecture, we feed OptiMathSAT some formula equivalent to:

4 $$\begin{aligned}{} & {} \ \forall \underline{\textbf{x}}. \left[ \begin{array}{ll} {(F(\underline{\textbf{x}}) \rightarrow \exists \underline{\textbf{a}}. (P_F(\underline{\textbf{x}},\underline{\textbf{a}} |\underline{\varvec{\theta }}) =0 ))\ \wedge }\\ (F(\underline{\textbf{x}}) \rightarrow \forall \underline{\textbf{a}}. (P_F(\underline{\textbf{x}},\underline{\textbf{a}} |\underline{\varvec{\theta }}) \ge 0 ))\ \wedge \\ (\lnot F(\underline{\textbf{x}}) \rightarrow \forall \underline{\textbf{a}}. (P_F(\underline{\textbf{x}},\underline{\textbf{a}} |\underline{\varvec{\theta }}) \ge g_{min} )) \wedge \\ \bigwedge _i(\theta _i \in [-4,4]) \wedge \bigwedge _{i,j}(\theta _{ij} \in [-2,1]) \end{array} \right] , \end{aligned}$$

asking to find the set of values of the $\theta$s satisfying (4) which maximizes the gap $g_{min}$. The result, if any, is a suitable $P_F(\underline{\textbf{x}},\underline{\textbf{a}} |\underline{\varvec{\theta }})$.

### Locally-structured embedding for large SAT problem

Encoding a Boolean formula $F(\underline{\textbf{x}})$ using the monolithic encoding shown in (4) presents several limitations. In practice, no more than 10 qubits can be considered if we directly use the formulation in Eq. (4), and recalling that some of them are required as ancillary variables, the set of Boolean formulas we can encode monolithically this way is quite limited.

To encode larger propositional problems, Bian et al.^21^ proposed a *divide-and-conquer* strategy. The original formula is first And-decomposed into smaller sub-formulae so that the penalty function $P_F(\underline{\textbf{x}},\underline{\textbf{a}} |\underline{\varvec{\theta }})$ for each subformula can be computed for some given placement. In particular, given a formula $F(\underline{\textbf{x}})$, we can And-decompose it as $F(\underline{\textbf{x}}):= \bigwedge _{k=1}^K F_k(\underline{\textbf{x}} ^k)$, so that each penalty function can be computed offline by OptiMathSAT. The *And-decomposition property*^21^ guarantees under some conditions that the penalty function of the original formula $F(\underline{\textbf{x}})$ can be easily obtained by summing up all the penalty functions from the subformulae: $P_F(\underline{\textbf{x}},\underline{\textbf{a}} |\underline{\varvec{\theta }}) = \sum _k P_{F_k}(\underline{\textbf{x}} ^k,\underline{\textbf{a}} ^k|\underline{\varvec{\theta }} ^k)$, where $g_{min}(F(\underline{\textbf{x}})) = min_k(g_{min}^k(F_k(\underline{\textbf{x}})))$. The penalty function $P_{F_k}(\underline{\textbf{x}^k},\underline{\textbf{a}^k} |\underline{\varvec{\theta }} ^k)$ of each sub-formula $F_k(\underline{\textbf{x}} ^k)$ is then mapped into a subgraph in the QA topology –e.g. one of the tiles in the Pegasus topology.

When two sub-formulae $F_i(\underline{\textbf{x}} ^i)$ and $F_j(\underline{\textbf{x}} ^j)$ share one (or more) Boolean variables *x*, we can (implicitly) rename one of the two occurrences into $x'$ and conjoin a chain of equivalences $x\leftrightarrow ... \leftrightarrow x'$ to them. (I.e., $F_i(...,x,...)\wedge F_j(...,x,...)$ can be (implicitly) rewritten into $F_i(...,x,...)\wedge F_j(...,x',...)\wedge (x\leftrightarrow ... \leftrightarrow x')$.) This corresponds to linking the corresponding qubits *x* and $x'$ in the penalty functions $P_{F_i}(\underline{\textbf{x}} ^i,\underline{\textbf{a}} ^i|\underline{\varvec{\theta }} ^i)$ and $P_{F_j}(\underline{\textbf{x}} ^j,\underline{\textbf{a}} ^j|\underline{\varvec{\theta }} ^j)$ by means of a *chain* of unused qubits used as ancillary variables, forcing all involved qubits to assume the same truth value, by using the *equivalence chain penalty function* $\sum _{(z, z') \in chain} (2 - 2zz')$ for the qubits in the chain, corresponding to the Boolean formula $x \leftrightarrow ...\leftrightarrow x'$ (here we consider the Pegasus extended ranges). The final penalty function is the sum of the penalty functions from the decomposition phase with those of the chains.

We refer the reader to Bian et al.^21^ for a more detailed description of these techniques.

## Methods: encoding binary multipliers into Pegasus quantum annealers

### Modular representation of a multiplier

**Figure 2:**
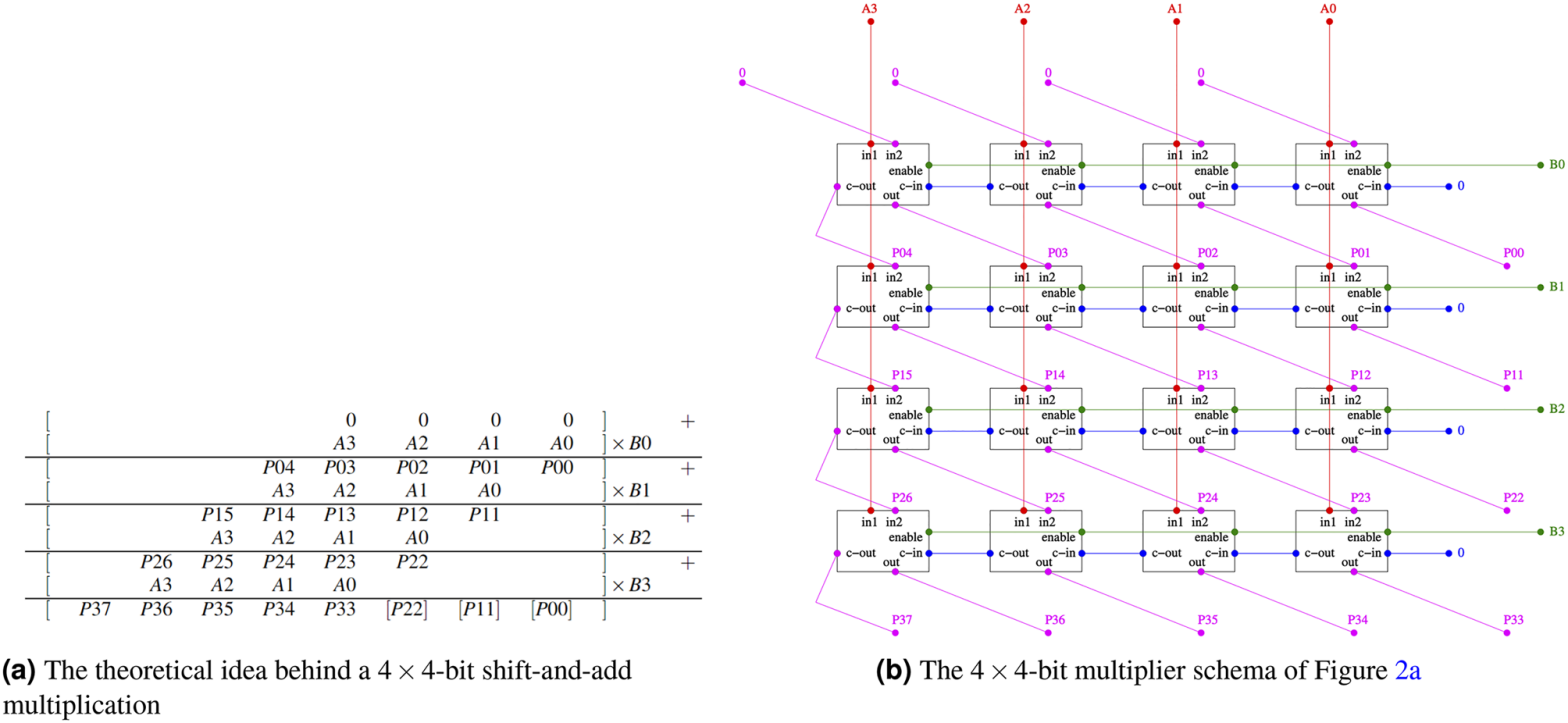
Details about the modularity of shift-and-add multipliers.

In a fashion similar to Bian et al.^21^, we developed a *modular* encoding of a shift-and-add multiplier, so that it could be easily extended for future larger quantum devices. To this extent, the binary-arithmetic computation of multiplications, as shown in Fig. 2a, is based on a module implementing a *Controlled Full-adder (CFA)*. The Boolean representation of a single CFA is:

$$\begin{aligned} CFA (in2, in1, enable, c\_in, c\_out, out) {\mathop {=}\limits ^{\text {\tiny def}}}&\big (c\_out \leftrightarrow ((c\_in \wedge ((enable \wedge in1) \vee in2)) \vee ((enable \wedge in1) \wedge in2)\big ) \\ \wedge&\big (out \leftrightarrow ((enable \wedge in1) \oplus in2 \oplus c\_in)\big ) \end{aligned}$$

The structure of a CFA includes four inputs: two operand bits (*in*1 and *in*2), a control bit (*enable*) and a carry-in bit $c\_in$. The output-carry bit $c\_out$ and the output *out* of a CFA are computed as is it typically done for classical full adder, the only difference being the the fact that the input *in*1 is enabled by the *enable* bit: when *enable* is true, the CFA behaves as a standard full adder; when *enable* is false, the CFA behaves as if *in*1 were false.

As shown in Fig. 2b, an $m\times n$-bit multiplier can be encoded using $m\cdot n$ CFAs as follows:

5 $$\begin{aligned} F_{P=A\times B}&= \bigwedge _{i=0}^{n-1}\bigwedge _{j=0}^{m-1} CFA (in2^{(i, j)}, in1^{(i, j)}, enable^{(i, j)}, c\_in^{(i, j)}, c\_out^{(i, j)}, out^{(i, j)}) \wedge \bigwedge _{(x, x') \in chains} (x \leftrightarrow x') \end{aligned}$$

where *chains* corresponds to the set of all the equivalence chains corresponding to the links between bits belonging to different CFAs, as in Fig. 2b (e.g. $(enable^{(i,j)}\leftrightarrow enable^{(i,j+1)}$).

### LSE-based encoding with qubit sharing, virtual chains, and alternating CFAs

A direct approach to building multipliers using multiple CFAs is to encode each CFA into a single Pegasus tile, using 2 of the 8 total qubits as ancillae. Once the penalty function for a single CFA has been obtained, we can embed them modularly and generate a grid of CFAs that simulates the multiplier. Since some qubits are shared among different CFAs, we must add equivalence chains to force the equality of the values of the corresponding qubits. First, the carry-out $c\_out$ qubit of a CFA placed into one tile must be linked to the carry-in $c\_in$ qubit of the CFA placed in the tile hosting the left CFA in the grid in Fig. 2b. The same applies to the output *out* of a CFA and the input *in*2 in the bottom-left CFA in Fig. 2b. Lastly, it is necessary to generate the qubits links corresponding to the long red vertical chain and the green horizontal chain in Fig. 2b, linking respectively the *in*1 and *enable* bits.

**Figure 3:**
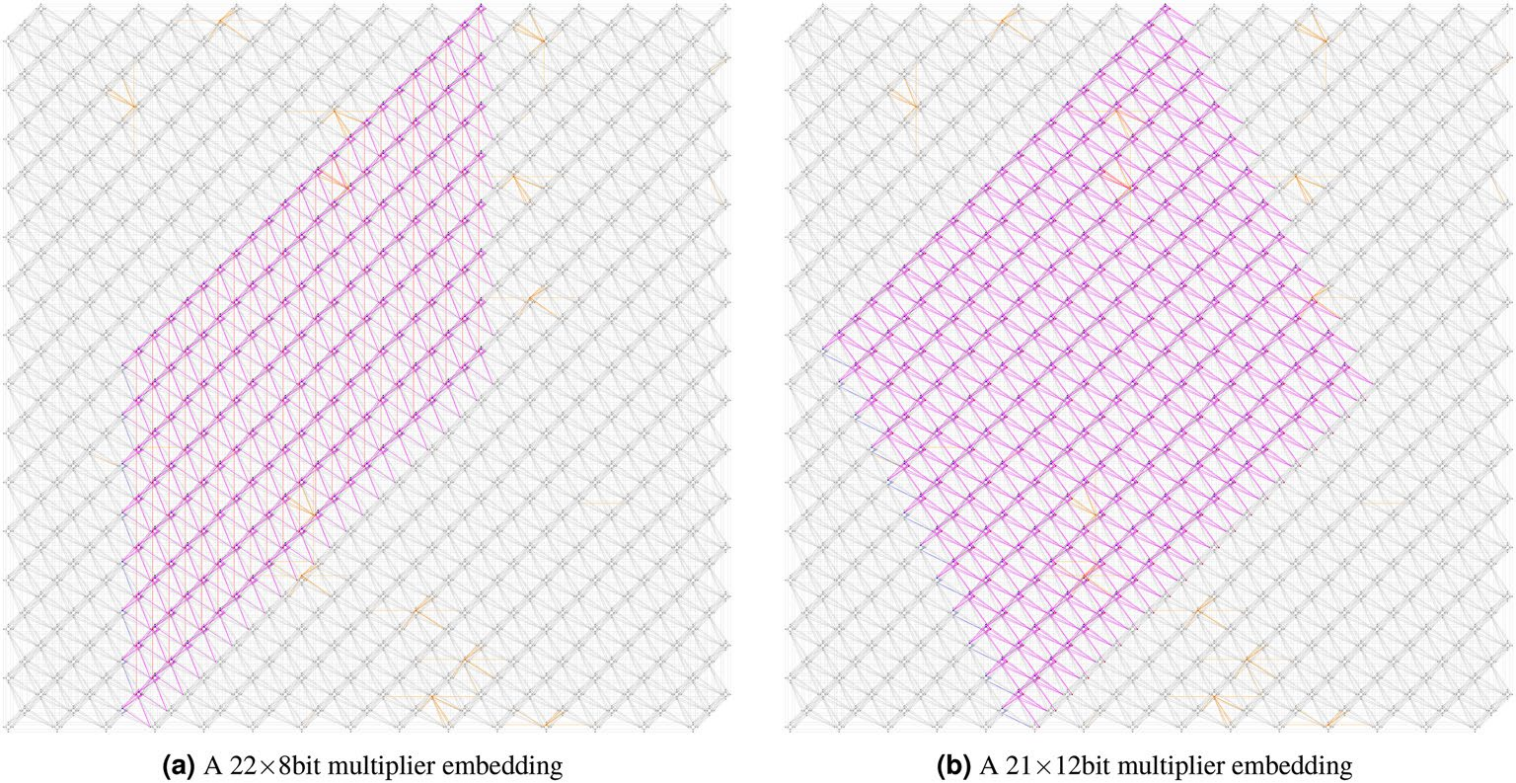
Modular encoding of binary multipliers on the D-Wave Pegasus topology.

In the Pegasus topology, each tile has some direct connections with the neighbor tiles along several directions (expressed in degrees counterclockwise with respect to the horizontal line): $0^\circ$, $90^\circ$, $45^\circ$, $120^\circ$ and $150^\circ$. Considering all these constraints, two macro-configurations for placing the CFA grid of Fig. 2b into a Pegasus architecture can be considered. In both configurations, due to the high number of inter-tile $45^\circ$ connections, the horizontal connections in Fig. 2b (the $c\_out-c\_in$ and *enable* links) are placed along the $45^\circ$ inter-tile connections. With the first configuration, in Fig. 3a, the input qubits *in*1 from vertically-aligned CFAs in the grid are connected by 90^∘^ inter-tile connections and the $out-in2$ links are connected via $120^\circ$ ones. This allows for fitting a 22 $\times$ 8-bit multiplier into the whole Pegasus topology. The second configuration, in Fig. 3b, differs from the first one by chaining the *in*1 qubits along 120^∘^ connections and the $out-in2$ links along 150^∘^ ones. Using diagonal chains has the main advantage to fit a larger 21 $\times$ 12-bit multiplier. Both configurations work modulo symmetries: for instance, encoding the grid of CFAs such that the input variable *in*1 is propagated bottom-up instead of top-down is feasible by slightly changing the qubits placement into the tile.

Unfortunately, an 8-qubit CFA encoding to replicate the two configurations described above turned out to be unfeasible in practice, because no such encodings can be generated. This fact is due to two main issues: (*i*) the low number of ancillae (only 2) available for encoding each CFA, which drastically reduces the chances of finding a suitable penalty function, and (*ii*) the absence of pairwise direct 45^∘^ couplings between the same qubits in the neighbor tiles, which prevents any direct implementation of the *enable* chain along the 45^∘^ direction. (A similar issue occurs also in the second macro-configuration of Fig. 3b for the the *in*1 bit along the 120^∘^ direction.)

To cope with these issues, we propose three novel techniques: *Alternating CFAs*, *Qubit sharing*, and *Virtual chaining*.

### Alternating CFAs

**Figure 4:**
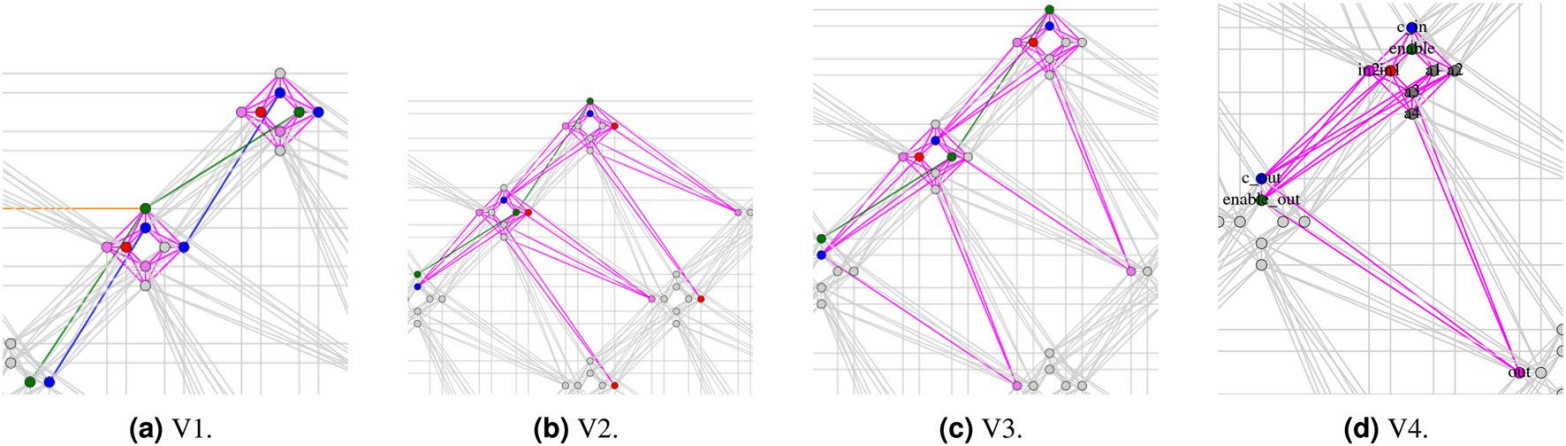
CFA structure for the four versions of multipliers.

To address the issue (ii) of missing couplings between qubits on the 45^∘^ direction, we propose to alternate two slightly-different CFAs in tiles along the 45^∘^ line. In particular, in Fig. 4b,c we make the OMT solver compute two different CFAS forcing *enable* to be positioned respectively in the first vertical qubit on the upper tile and the third horizontal qubit in the 45° bottom-left tile. Such qubits are pairwise directly coupled, allowing thus a chain for *enable* qubit along the 45° direction (the green links). We stress the fact that the two different CFA encodings are not guaranteed to have the same gap $g_{min}$, and that different placements leading to different $g_{min}$ values typically may negatively affect the annealing process.

### Qubit sharing

To address the issue (i) of the low number of ancillae, we propose a technique to *share qubits between neighboring tiles*. Rather than connecting two qubits from different CFAs with an equivalence chain, we suggest utilizing a single qubit that is *shared* between the two CFAs. This means that the qubit will be used for the encoding of one CFA as an output variable and as an input variable for the subsequent CFA. This approach leads to partially-overlapping CFAs and the extra qubit can be used as an ancillary variable to increase the minimum gap of each CFA. Consider the schema in Fig. 4d. The encoding of each CFA involves not only the 8 qubits of its tile but also the 3 qubits of neighbor tiles. In particular, the carry-out $c\_out$ is placed on the same qubit as the carry-in $c\_in$ of the next 45° bottom-left tile –corresponding to the left CFA in Fig. 2b—and the *out* qubit is placed in the same qubit of the *in*2 of the next bottom-right 120° tile –corresponding to the lower-right CFA in Fig. 2b. The same idea applies also to the schemata in Fig. 4b,c. (The role of the $enable\_out$ qubit in Fig. 4d will be explained later.)

Notice that, since the global penalty function is the sum of the penalty functions of all CFAs plus these of all the equivalence chains, the value of the bias for the shared qubit in the global penalty function is the sum of these two qubits with different roles in the two penalty functions of the two sharing CFAs. (E.g., the bias of the qubit which is a $c\_out$ for one CFA and a $c\_in$ for another CFA is the sum of the $c\_in$ and $c\_out$ biases of a CFA encodings.) Thus, to generate penalty functions for the CFAs that allow qubit sharing, we introduce additional constraints to the OMT formulation in (4). In particular, we add an arithmetical constraint to force the sum of the biases of the shared qubits from two CFAs to fit in the bias range, thus simulating their over-imposition (e.g., we add a constraint like $(\theta _{c\_{in}} + \theta _{c_{out}} \in [-4, 4])$). In fact, if the final bias values did not fit into the range, then the D-Wave encoders would automatically rescale all values of biases and couplings, reducing the $g_{min}$ value and thus negatively affecting the probability of reaching a global minimum.

### Virtual chaining

The concept of qubit sharing can be exploited to simulate the existence of equivalence links when physical connections are missing, providing another solution to issue (ii). Consider the CFA encoding in Fig. 4d and the *enable* logical variable. Its truth value is shared by all CFAs belonging to the same row in the grid so that all the *enable* qubit of each CFA should be connected by an equivalence chain with the *enable* qubit of the 45^∘^ bottom-left CFA. Unfortunately, there is no arc linking pairwise the respective qubits of the tiles along this direction.

In such cases, two qubits that are intended to hold the same truth value but lack a direct coupling can be *virtually chained* by using the links with the common neighbors. This is performed by extending the encoding as follows:

- Create a new virtual logical variable (i.e. $enable\_out$) to be placed in the qubit in the neighbor tile corresponding to the variable we want to chain virtually (i.e. *enable*);
- Extend the formula defining a CFA by conjoining the equivalence constraint between the chained and the virtual variables (i.e., $CFA' (in2, in1, enable, c\_in, c\_out, out,enable\_out) {\mathop {=}\limits ^{\text {\tiny def}}} CFA (in2, in1, enable, c\_in, c\_out, out) \wedge (enable \leftrightarrow enable\_out)$;
- Build the penalty function of $CFA'$ instead of *CFA* by applying qubit-sharing also to *enable* and $enable\_out$.

It should be noted that if two directly-connected qubits are both involved in qubit sharing (i.e. $c\_in$ and *enable*), then also the respective coupling is shared by the two CFAs. Therefore an arithmetic constraint must be added to force the sum of the two couplings to be in the coupling range (i.e. $(\theta _{c\_in,enable}+\theta _{c\_out,enable\_out}\in [-2,1])$).

### Comparing different multiplier configurations

Overall, exploiting Alternating CFAs, qubit sharing, and Virtual chaining made it possible for us to generate four multiplier configurations, which are summarized in Table 1. Versions V1, V3 and V4 allow for implementing the 22 $\times$8-bit schema of Fig. 3a, whereas version V2 allows for implementing the 21 $\times$ 12-bit schema of Fig. 3b. Versions V2, V3 and V4 correspond to the encodings in Fig. 4b–d respectively.

In particular: by exploiting *Alternating CFAs*, with versions V1, V2 and V3 (Fig.  4a–c), we could implement an *enable* chain along the 45^∘^ diagonal, and with version V1 (Fig.  4b) an *in*1 chain along the 120^∘^ diagonal; by exploiting *Qubit sharing*, with versions V2, V3, V4 (Fig. 4b–d), we have saved two qubits, which we could use as ancillae, improving also the quality of the encodings and their gap $g_{min}$; by exploiting *Virtual chaining*, with V4 (Fig. 4d), we could implement a virtual chain for the *enable* qubit along the 45^∘^ diagonal; with V2 (Fig. 4b) we could implement a virtual chain for the *in*1 qubit along the 120^∘^ diagonal.

Version V1 (Fig. 4a) implements the 22 $\times$ 8-bit macro-configuration of Fig. 3a and relies exclusively on alternating CFAs, linking inter-tile qubits only by physical chains. Although alternation allowed the production of an actual encoding, which was not possible otherwise, without qubit sharing only two ancillae were available, producing two alternating configurations with different and very low gaps: 1 and $\frac{4}{9}$. These numbers are way lower than the gap used for chains, the annealers tend to be stuck on local minima since changing the spin of chained qubits becomes difficult.

Version V2 (Fig. 4b) implements the 21 $\times$ 12-bit macro-configuration of Fig. 3b with alternating CFA encodings, using a virtual chain for implementing the *in*1 chain along the 120^∘^ direction, and qubit sharing for the $c\_in-c\_out$ (the blue qubits) and $out-in2$ (the magenta qubits) connections, which saves two qubits and allows for 4 ancillae. This allows us to improve significantly the gaps to 2 and $\frac{4}{3}$ respectively. Nevertheless, the two CFAs have different $g_{min}$, which negatively affects the global gap (which is thus $\frac{4}{3}$) and thus the overall performances of the annealer.

Version V3 (Fig. 4c) instead implements the 22 $\times$ 8-bit macro-configuration of Fig. 3a with alternating CFA encodings, using a physical 90^∘^ *in*1, also using qubit sharing for the $c\_in-c\_out$ and $out-in2$ connections, allowing 4 ancillae. With this configuration, we obtain two CFAs with identical gap 2, which is a significant improvement. Nevertheless, having two physical chains for two different variables (*enable* and *in*1) affects the annealer’s performances: the longer the chains, the more difficult is for the quantum system to flip all values of the chained qubits and escape a minimum.

Version V4 (Fig. 4d) also implements the 22 $\times$ 8-bit macro-configuration of Fig. 3a, but uses only one CFA encoding of gap 2. This is achieved by exploiting not only qubit sharing for the $c\_in-c\_out$ and $out-in2$ connections, but also virtual chaining for implementing the *enable* chain, whereas *in*1 is physically chained vertically. By using a single CFA and having only one physical chain rather than two, most of the issues affecting annealing in the previous cases is solved, thus the optimization of the penalty function by the QA turns out to be more effective. Consequently, all experiments in the subsequent section employ version V4.

**Table 1:** Comparison of the four multipliers obtained through qubit sharing and virtual chaining.

Multiplier version	V1	V2	V3	V4
Multiplier Max. Size	22 $\times$ 8	21 $\times$ 12	22 $\times$ 8	22 $\times$ 8
# of ancillae per CFA	2	4	4	4
# of different CFA encodings	2	2	2	1
Gap of CFA penalty functions	(1, $\frac{4}{9}$)	(2,$\frac{4}{3}$)	(2,2)	2
Connection $in1(i,j)-in1(i+1,j-1)$	Chain (90^∘^)	Virtual chain (120^∘^)	Chain (90^∘^)	Chain (90^∘^)
Connection $enable(i,j)-enable(i,+1)$	Chain (45^∘^)	Chain (45^∘^)	Chain (45^∘^)	Virtual chain (45^∘^)
Connection $c\_in(i,j)-c\_out(i,j+1)$	Chain (45^∘^)	Qubit sharing	Qubit sharing	Qubit sharing
Connection $out(i,j)-in2(i+1,j-1)$	Chain (45^∘^)	Qubit sharing	Qubit sharing	Qubit sharing

## Methods: solving prime factorization on D-wave advantage 4.1 system

The results presented in the previous section do not account for the actual limitations of quantum annealers. In particular, due to hardware faults, some of the qubits, and some connections between them are inactive and cannot be tuned during annealing. These inactive nodes and connections, referred to in the literature as *faulty qubits* and *faulty couplings* respectively, are spread all around the entire architecture, and are marked in orange in Fig. 3a,b for the D-Wave Advantage 4.1 annealer, which we have used in all our experiments in this paper. Therefore, although it is theoretically possible to create multipliers up to $21\times 12\text { bits}$ or $22\times 8\text { bits}$, these hardware constraints compel us to test smaller multipliers to avoid faulty qubits and couplings. An empirical evaluation of possible placements of multipliers into the Advantage 4.1 system leads us to determine an area of the architecture with no faulty nodes nor couplings that is suitable for being tested, capable of embedding a multiplier of maximum size $17\times 8\text {bits}$ with the configuration of Figs. 3a and 4d. All the experiments in this section will consider these hardware limitations. Also, the experimental evaluation reported in this section was constrained by the limited amount of QPU time on the Advantage 4.1 annealer we were given access to (600 seconds per month). As a consequence, no extensive statistical evaluation can be made on the experiments, so that no standard deviation has been inferred on the outcomes. Also, it could be the case that there may have been some lucky (and likewise unlucky) shots among the tests.

### Initializing qubits

To factor a specific integer, it is necessary to initialize several qubits within the multiplier embedding: all qubits associated with the output bits need to be initialized to represent the target number for factorization —e.g., if the output [*P*37...*P*00] of the 4 $\times$ 4-bit multiplier in Fig. 2a,b is forced to 00100011 (i.e. 35), then the corresponding qubits are initialized respectively to $\{{-1,-1,1,-1,-1,-1,1,1}\}$; additionally, the variables $c\_in$ and *in*2 on the most external CFAs should be forced to be 0, as depicted in Fig. 2b, so that their corresponding qubits should be initialized to $-1$.

D-Wave Advantage interface provides an API, the $fix\_variables()$ function, which allows us to impose desired values on the qubits of the underlying architecture. This function operates by substituting the values of the qubits into the penalty function and subsequently rescaling the resulting penalty function to ensure all coefficients fall within the limited ranges of biases and couplings, possibly resulting into a lower $g_{min}$. For instance, if we have the penalty function $P_F(\underline{\textbf{x}} |\underline{\varvec{\theta }}) = 2 + 4x_1 + x_2 + x_1x_2$ and we set $x_2$ to 1, then the penalty function becomes $P'_F(\underline{\textbf{x}} |\underline{\varvec{\theta }}) = 2 + 4x_1 + 1 + x_1 = 3 + 5x_1$, which is then rescaled into $12/5 + 4x_1$ by multiplying it by a 4/5 factor in order to fit the bias of $x_1$ into the $[-4,4]$ range, thus reducing $g_{min}$ by multiplying it the same 4/5 factor. On the one hand, this substitution simplifies the penalty function by removing one binary variable; on the other hand, it can hurt the minimal gap due to coefficient rescaling.

To cope with the latter problem, we propose an alternative method to initialize qubits on a quantum device. We can partially influence the quantum annealer to set a specific truth value for a qubit by configuring *flux biases*^24^. In particular, if we want to impose the value $s_i\in \{-1,1\}$ on a qubit, we set the flux bias for that qubit as $\phi _i = 1000\phi _0s_i$, where $\phi _0$ is the default annealing flux-bias unit of the DWave system 4.1, whereas 1000 is an empirical value we choose based on our experience.

The experiments suggested a further minor improvement in the CFA encoding. Since there may be more than one penalty function with the optimum value of $g_{min}$, we make a second call to an OMT solver in which we fix $g_{min}$ and ask the solver to find a solution which also minimizes the number of those falsifying assignments which make the penalty function equal to $g_{min}$. The intuition here is to minimize the possibility of the annealer to get excited from ground states to first excited un-satisfying states. (Hereafter we refer as “CFA1” the CFA encoding obtained with this improvement and as “CFA0” the basic one.)

In Table 2 (left) we compare the performances of the two initialization techniques on small prime factorization problems, with the annealing time $T_a$ set to $10\mu s$. The column labeled $\#(P_F = 0)$ reports how many occurrences of 0-energy samples are obtained out of 1000 samples. We noticed that flux biases (with CFA1) outperform the native API, having a higher probability of reaching the global minimum. All the experiments from now on assume qubit initialization is done by tuning flux biases.

**Table 2:** Results of standard forward annealing to solve prime factorization.

Size	Input *N*	CFA0	CFA1		
		$\#(P_F = 0)$	$\#(P_F = 0)$		
3 $\times$ 3	25 (5 $\times$ 5)	161	136		
	35 (5 $\times$ 7)	389	951		
	49 (7 $\times$ 7)	450	997		
4 $\times$ 4	121 (11 $\times$ 11)	17	0		
	143 (11 $\times$ 13)	40	67		
	169 (13 $\times$ 13)	31	5		
5 $\times$ 5	289 (17 $\times$ 17)	5	0		
	323 (17 $\times$ 19)	2	0		
	361 (19 $\times$ 19)	1	3		
	391 (17 $\times$ 23)	6	9		
	437 (19 $\times$ 23)	17	0		
	493 (17 $\times$ 29)	3	2		
	527 (17 $\times$ 31)	21	37		
	529 (23 $\times$ 23)	5	8		
	551 (19 $\times$ 29)	0	4		
	589 (19 $\times$ 31)	16	52		
	667 (23 $\times$ 29)	0	105		
	713 (23 $\times$ 31)	11	138		
	841 (29 $\times$ 29)	5	7		
	899 (29 $\times$ 31)	17	343		
	961 (31 $\times$ 31)	1	338		

Size	Input *N*	$\#(P_F = 0)$	Size	Input *N*	$\#(P_F = 0)$
7 $\times$ 7	10,033 (127 $\times$ 79)	0	8 $\times$ 8	49,447 (251 $\times$ 197)	0
	10,541 (127 $\times$ 83)	**1**		49,949 (251 $\times$ 199)	0
	11,303 (127 $\times$ 89)	0		52,961 (251 $\times$ 211)	0
	12,319 (127 $\times$ 97)	0		55,973 (251 $\times$ 223)	0
	12,827 (127 $\times$ 101)	1		56,977 (251 $\times$ 227)	0
	13,081 (127 $\times$ 103)	2		57,479 (251 $\times$ 229)	0
	13,589 (127 $\times$ 107)	10		58,483 (251 $\times$ 233)	0
	13,843 (127 $\times$ 109)	0		59,989 (251 $\times$ 239)	2
	14,351 (127 $\times$ 113)	0		60,491 (251 $\times$ 241)	0
	16,129 (127 $\times$ 127)	7		63,001 (251 $\times$ 251)	0

We used 1000 samples for each annealing step. *Left:* Comparison of the two initialization techniques on prime factorization of small numbers, with $T_a = 10 \mu s$. *Right*: Prime factorization of the 10 biggest 7 $\times$ 7 and 8 $\times$ 8 numbers configuring flux biases, with $T_a=10$ μs.

### Exploiting thermal relaxation

In order to test the limits of the flux-bias initialization, we applied it to factoring the 10 largest numbers of 7 $\times$ 7 and 8 $\times$ 8 bits with the same annealing time as the previous experiments ($T_a=10\mu s$.) The results, reported in Table 2 (right), suggest that the success probability of getting a solution for 16-bit numbers is almost null. Increasing the annealing time $T_a$, however, would probably not significantly increase the success probability; to further improve the solving performances, we investigate the effectiveness of *thermal relaxation*^25^ on solving our problems. This technique is integrated into the DWave system by introducing a *pause* $T_p$ at a specific point $S_p$ during the annealing process, with $S_p\in [0,1]$. We tested it to solve 8 $\times$ 8, 9 $\times$ 8 and 10 $\times$ 8-bit factorization problems.

**Table 3:** Results about prime factorization solved through QA, exploiting thermal relaxation.

Size	Input *N*	$S_p$	$min(P_F)$	#$(P_F=0)$	Size	Input *N*	$S_p$	$min(P_F)$	#$(P_F=0)$	Size	Input *N*	$S_p$	$min(P_F)$	#$(P_F=0)$
8 $\times$ 8	49,447 (251 $\times$ 197)	0.38	0.000	1	9 $\times$ 8	100,273 (509 $\times$ 197)	–	4.083	0	10 $\times$ 8	201,137 (1021 $\times$ 197)	–	6.167	0
	49,949 (251 $\times$ 199)	–	4.083	0		101,291 (509 $\times$ 199)	–	8.083	0		203,179 (1021 $\times$ 199)	–	8.000	0
	52,961 (251 $\times$ 211)	–	6.000	0		107,399 (509 $\times$ 211)	–	4.000	0		215,431 (1021 $\times$ 211)	–	6.083	0
	55,973 (251 $\times$ 223)	0.33	0.000	6		113,507 (509 $\times$ 223)	–	8.083	0		227,683 (1021 $\times$ 223)	0.34	0.000	1
	56,977 (251 $\times$ 227)	0.33	0.000	1		115,543 (509 $\times$ 227)	0.33	0.000	1		231,767 (1021 $\times$ 227)	–	8.083	0
	57,479 (251 $\times$ 229)	0.33	0.000	3		116,561 (509 $\times$ 229)	–	6.000	0		233,809 (1021 $\times$ 229)	–	8.000	0
	58,483 (251 $\times$ 233)	–	6.083	0		118,597 (509 $\times$ 233)	–	4.000	0		237,893 (1021 $\times$ 233)	–	6.000	0
	59,989 (251 $\times$ 239)	0.33	0.000	43		121,651 (509 $\times$ 239)	0.33	0.000	1		244,019 (1021 $\times$ 239)	–	6.250	0
	60,491 (251 $\times$ 241)	0.38	0.000	1		122,669 (509 $\times$ 241)	–	8.167	0		246,061 (1021 $\times$ 241)	–	6.167	0
	63,001 (251 $\times$ 251)	–	2.000	0		127,759 (509 $\times$ 251)	0.36	0.000	1		256,271 (1021 $\times$ 251)	0.35	0.000	2

Size	Input *N*	Forward annealing		Reverse annealing										
		$S_p$	$min(P_F)$	$S'_p$	$P_F$	$\Delta HAM$	#$(P_F=0)$							
8 $\times$ 8	49,949 (251 $\times$ 199)	0.50	2.000	0.33	0.000	233	7							
	52,961 (251 $\times$ 211)	0.35	2.000	0.41	0.000	177	1							
	58,483 (251 $\times$ 233)	0.33	2.083	–	4.000	144	0							
	63,001 (251 $\times$ 251)	0.51	2.000	0.35	0.000	168	4							
9 $\times$ 8	100,273 (509 $\times$ 197)	0.36	4.000	–	4.083	198	0							
	101,291 (509 $\times$ 199)	0.44	4.000	–	4.000	2	0							
	107,399 (509 $\times$ 211)	0.51	4.000	–	4.000	79	0							
	113,507 (509 $\times$ 223)	0.38	2.000	–	2.000	71	0							
	116,561 (509 $\times$ 229)	0.36	4.000	0.37	0.000	98	35							
	118,597 (509 $\times$ 233)	0.33	2.000	–	4.000	201	0							
	122,669 (509 $\times$ 241)	0.48	4.083	0.36	0.000	129	7							
10 $\times$ 8	201,137 (1021 $\times$ 197)	0.38	2.000	–	2.000	6	0							
	203,179 (1021 $\times$ 199)	0.34	4.000	–	4.083	218	0							
	215,431 (1021 $\times$ 211)	0.4	4.000	–	4.000	228	0							
	231,767 (1021 $\times$ 227)	0.33	2.083	–	4.083	201	0							
	233,809 (1021 $\times$ 229)	0.39	4.083	–	6.000	112	0							
	237,893 (1021 $\times$ 233)	0.46	2.083	–	2.083	2	0							
	244,019 (1021 $\times$ 239)	0.48	2.000	–	4.000	137	0							
	246,061 (1021 $\times$ 241)	0.34	4.000	–	2.083	142	0							

We used 1000 samples for each annealing step. *Top:* Prime factorization of 8 $\times$ 8, 9 $\times$ 8 and 10 $\times$ 8-bit numbers, with $T_a = 10$ μs and pause $T_p = 100$ μs. *Bottom:* Results of performing reverse annealing on the problem instances not solved in Table 3 (top), with $T_a = 10$ μs and $T_p = 10$ μs. The label $\Delta HAM$ reports the Hamming distance between the forward annealing lowest energy sample and the reverse annealing lowest energy sample.

In the experiments, the pausing time $T_p$ was set to $100$ μs, whereas the pause point $S_p$ is selected in the set $\{0.33, 0.34, \ldots , 0.51\}$ and tested in ascending order until the ground state is found.

The results illustrated in Table 3 (top), if compared with these in Table 2 (right) indicate the positive impact of thermal relaxation. Ground states were successfully reached for some 18-bit numbers (the largest being 256271), although challenges persist with most numbers of that size.

### Exploiting quantum local search

For the factorization problems in Table 3 (top) that did not end up in the global minimum, we further exploited *quantum local search*, consisting of refining a sub-optimal state to reach the global minimum. Quantum local search is implemented in the DWave system by mean of *reverse annealing* (RV)^26^. The annealer is initialized in a local minimum, whereas the annealing process starts from $s=1$ moving towards $s'=0$ and then returning back to $s=1$. We remark that reverse annealing admits pauses during the process: in this case, the system pauses for $T_p$ microseconds at a middle point $s'=S'_p$.

In our experiments, we chose the lowest-energy state from Table 3 (top) as the initial state of RV. If multiple lowest-energy samples are obtained with different $S_p$ values, we pick the one whose pause is performed later. The pause points for RV were tested in decreasing order (in opposition to forward annealing when we opted for the ascending order) until a ground state was found. The results are reported in Table 3 (bottom). We observe that reverse annealing, enhanced by thermal relaxation, helps in solving up to 9 $\times$ 8-bit factorization problems. We also reported the Hamming distance $\Delta HAM$ between the lowest-energy state from forward and reverse annealing, showing how much a sample moved from one minimum to another, possibly a ground state.

For the instances that still failed to reach a solution, we investigated the impact of different pause lengths for RV to find ground states. The main observation from this additional analysis is that, given a low-energy initial state: (*i*) increasing the pause length and performing the pause at a late annealing point can help reverse annealing in jumping larger Hamming distances; (*ii*) increasing the pause length and triggering the pause at early annealing points cannot make RV move even farther. From these observations, we could imply that if the initial state of a reverse annealing process is very far from the ground state, it could be hard to reach the global minimum by only increasing the pause length. However, the local minimum used for the initial state of RV, which is obtained by standard annealing, tends to be highly excited (i.e., with high energy and very far from the ground state), as the problem size increases.

In the next section, we follow the *iterated reverse annealing*^27^ approach, which was studied numerically in a closed-system setting, and propose an iterative strategy for the DWave system to solve bigger problems. The goal is to converge to a low-energy state that can be used as the initial state for single-iteration RV to reach the global minimum with an effective pause $T_p$.

### Solving prime factorization with iterated reverse annealing (IRV)

**Table 4:** Result about IRV.

Size	Input *N*	#	$T_p$	$S_p$	$min(P_F)$	$min(P_F)_{new}$	$(HAM, \Delta HAM, HAM_{new})$
12 $\times$ 8	1,027,343 (4093 $\times$ 251)	1	1	0.31	10.167	4.000	(263, 142, 151)
		2	1	0.38	10.167	4.083	(128, 122, 58)
		3	100	0.38	4.083	0[**2**]	(58, 58, 0)
14 $\times$ 8	4,111,631 (16381 $\times$ 251)	1	1	0.35	18.167	8.083	(290, 353, 249)
		2	1	035	16.000	6.000	(273, 219, 240)
		3	50	0.37	10.000	2.083	(277, 280, 85)
		4	10	0.38	2.083	0[**67**]	(85, 85, 0)
16 $\times$ 8	16,445,771 (251 $\times$ 65521)	1	1	0.34	18.333	6.083	(, 294,)
		2	10	0.35	10.000	4.000	(, 374, )
		3	50	0.36	6.083	4.167	(, 292, )
		4	100	0.39	4.167	4.000	(, 8,)
13 $\times$ 8	2,055,941 (8191 $\times$ 251)	1	100	0.38	14.083	6.083	(204, 185, 55)
		2	100	0.42	6.083	0[**216**]	(55, 55, 0)
14 $\times$ 8	4,111,631 (16,381 $\times$ 251)	1	200	0.39	16.167	6.083	(164, 178, 136)
		2	200	0.44	6.083	0[**467**]	(136, 136, 0)
15 $\times$ 8	8,219,999 (32,749 $\times$ 251)	1	1	0.4	20.333	12.167	(279, 126, 217)
		2	100	0.43	12.167	8.000	(217, 180, 277)
		3	200	0.43	8.000	6.000	(277, 65, 282)
		4	200	0.44	6.000	4.083	(282, 247, 71)
		5	200	0.43	4.083	0[**329**]	(71, 71, 0)

The label $\Delta HAM$ reports the Hamming distance between the forward annealing lowest energy sample and the reverse annealing lowest energy sample. The labels *HAM* and $HAM_{new}$ report the Hamming distance of respectively the starting point and the lowest energy sample of that iteration with respect to the ground state. The bold number near 0 reports how many samples reached 0 energy for that iteration, out of 1000. *Top:* Results of the original IRV algorithm. For the 16 $\times$ 8 problem, since no ground state has been retrieved, no comparison of Hamming distances with the ground state is provided (thus *HAM* and $HAM_{new}$ are left empty). *Bottom:* Results of the IRV variant that focuses on longer pauses.

In general, we assume that starting reverse annealing from a state that is close to the ground state could be beneficial in finding the solution. We remark, however, that we have no prior knowledge of the solution. To cope with this missing information, we assumed that a low-energy state may be closer to the ground state and our proposal is built on top of this assumption.

The IRV strategy starts by running a standard forward annealing process, with thermal relaxation disabled. The obtained lowest-energy state is selected as the starting point for the subsequent iterations of the algorithm. At each iteration of the IRV, we execute a batch of RV processes, with several pause lengths $T_p$ and pausing points $S_p$ taken into consideration, until we obtain a lower-energy space. The *lower-energy space* refers to the set of lower-energy states retrieved in one iteration whose energy is *below* the starting point. Once that space has been retrieved, we check if there is a ground state in that space: when this happens, we have the solution for the problem and we stop the entire procedure; otherwise, this procedure is iterated until the system finds the ground state or hits a certain number of iterations.

It is not trivial to determine how long a pause should be and when to trigger it for the intermediate iterations to gradually approach the ground state. Based on the previous observations,

we chose a set of pause lengths e.g., $\{1, 10, 30, 50, 100\}\mu s$ and a set of pause point, e.g., $\{0.46,\ldots , 0.33\}$, adapting those parameters

to the initial states of this iteration. We tested IRV on the DWave Advantage System 4.1 by trying to factorize the numbers 1,027,343, 4,111,631, and 16,445,771 using respectively a 12 $\times$ 8, 14 $\times$ 8, and 16 $\times$ 8-bit multiplier. The experiments consider the assumptions discussed in the previous paragraphs, a further analysis of these conditions is left as future work. Table 4 (top) reports the successful search paths of IRV in finding the ground state, demonstrating that IRV is effective in reaching a solution even from an excited state very far away from the minimum, by approaching it gradually. We highlight that from our experiments it was impossible for standard reverse annealing to factor 4,111,631 even with a $600\mu s$ pause.

We also propose a variant of the IRV strategy discussed above. From the failed factorization of 16,445,771, we noticed that the last iteration got stuck in the local minimum even with a pause of $100\mu s$. To cope with this issue, we opted to focus on triggering long distances. This is done by increasing the pause length at each iteration, i.e., $T_p\in \{100, 200\}\mu s$. Correspondingly, we simplify the choice of the starting state for an iteration, choosing a lower energy state as the initial state of each iteration. The experimental results shown in Table 4 (bottom) demonstrate the improvement of this variant of IRV, in terms of fewer iterations required to reach the solution, at the cost of more QPU time.

Notice that in the case of the 23-bit number, 8,219,999, we use a pause of $1\mu s$. This is due to the fact the initial state is highly excited and a $1 \mu s$ pause can still trigger a relatively long distance, saving QPU time. According to the results in Table 4 (bottom), we highlight how the fourth iteration highly benefits from the long pause. Despite starting from a local minimum that is very far away from the solution, the long pause enables RV to travel long Hamming distances and reach a local minimum closer to our solution. This closer state provides a good initial state for the last-iteration RV to find the solution successfully.

Overall, exploiting all the encoding and solving techniques described in this paper, $8,219,999=32,749\!\times \!251$ was the highest prime product we were able to factorize. To the best of our knowledge, this is the largest number that was ever factorized by means of quantum annealing.

## Discussion

### A comparative analysis

#### About QA methods

In contrast to existing methodologies for solving prime factorization through quantum annealing^16–18^, which hing upon *global embeddings*, our approach introduces a novel modular encoding paradigm by adopting a strategy which is based on *local embedding*^20,21^.

In global embedding strategies, each problem instance undergoes a pre-processing phase and generates a penalty function that could be mapped only into unconstrained sub-graphs. Then, in order to map it into the constrained graph of quantum-device architecture, an additional step of *minor embedding* must be performed, which is provided by the D-Wave QA classical API. We remark that since the minor-embedding problem is NP-complete^28,29^, this step may be computationally demanding, and the classical computational effort to perform it grows with the size and the complexity of the problem instance.

Our method, instead, relies on local embedding: we have computed offline and once forever via OMT an encoding of a single CFA which is aligned with the Pegasus topology; to encode a $n\times m$-bit multiplier, we simply replicate it $n\times m$ times into a matrix structure, eliminating the need for minor embedding; then each PF instance is produced by simply forcing the values of the output qubits of the multiplier. We stress the fact that, due to the modularity of the encoding and unlike previous approaches, the size of the PF problems we can encode into a Pegasus QA scales-up automatically and effortlessly with the growth of the qubit number in future chips.

#### About hybrid methods

Despite many papers using the expression “hybrid classical-quantum”, there seems to be no universally-agreed definition of this concept. In this paper, by “hybrid classical-quantum” methods for solving computationally-hard search problems we mean methods in which, for each problem instance, the search effort is shared between quantum devices and classical search procedures. Therefore we have called “hybrid classical-quantum” the following methods.

- The D-Wave hybrid Classical-QA tool^30^, which is used in^19^, alternates the usage of D-Wave’s QAs on (smaller) subproblems, which result from a partitioning of the original (bigger) problem, with classical tabu search engines, which are used to combine these partial solutions and to refine them into a final global solution. Therefore, the computationally-hard search effort is shared between the quantum annealer and classical search procedures.
- The variational methods in^11,12^ heavily rely on the computational effort of classical search procedures optimizing the quantum-circuit parameters. In particular, given the input number, a measurement is sampled, yielding one of the possible outcomes from the quantum circuit. The measurement is then used to tune the parameters of the quantum circuit by invoking classical optimization algorithms. This adjustment continues iteratively until the parameters converge to values that minimize the cost function. Similarly to the previous case, the interleaving of quantum computation and classical optimization has a huge role in solving the factorization problem.

Our method, instead, integrates iterative quantum processes without relying on external classical search steps, and without using classical computing to enhance convergence or to refine intermediate states. Classical computing is only involved in interfacing with the quantum annealers, utilizing the output of one iteration as the input for the subsequent one based on predefined heuristics, using a predefined amount of iterations. To this extent, our method does not require any significant computational effort from any classical piece of software, and as such we do not refer to it as a hybrid method.

### Contextualization within the field

Despite in principle Shor’s algorithm suggests a theoretical quantum supremacy for PF problems, in practice the effectiveness of quantum techniques for PF is still far from that of standard algorithms run on classical computers, like the number field sieve^1^. Indeed the practical success of Shor’s algorithm is restricted by the scale of the gate-based quantum computers currently developed, like the IBM and Google devices, which is still very limited. A similar problem applies to the variational approaches, where only results for very specific biprime numbers have been obtained.

Quantum annealers are instead specialized quantum devices able to perform specific optimization and sampling tasks. One fundamental benefit of QAs with respect to classical optimization techniques, like simulated annealing, is the exploitation of effects like quantum tunneling to escape local minima^15^. On the one hand, a theoretical analysis of the running times required by the quantum-annealing hardware to factor integers is currently lacking in the literature, so that it is not currently possible to speak of any theoretical quantum speedup for QA-based PF algorithms. On the other hand, QAs are technologically significantly ahead of gate-based quantum computing in terms of qubit number, and D-Wave QAs have grown in size with a Moore-like exponential trend since their early stages^21^, currently reaching 5760 qubits.

To this extent, unlike with previous QA-based approaches to PF, our technique allows for encoding effortlessly arbitrarily-large multipliers and PF instances into a Pegasus architecture, being limited only by the size of current QA chips. Also, our technique could be used as an intermediate step inside the hybrid classical-QA methods^19,30^.

## Conclusions and future work

In this paper, we have proposed a novel approach to prime factorization by quantum annealing. Our contribution is twofold.

First, we have presented a novel modular *encoding* of a binary multiplier circuit into the Pegasus architecture of the most recent D-Wave QA devices. The key to success was a compact encoding of a controlled full-adder sub-circuit into an 8-qubit module in the Pegasus topology, which we synthesized offline by means of Optimization Modulo Theories. This allows us to encode up to a 21 $\times$ 12-bit multiplier (resp. a 22 $\times$ 8-bit one) into a 5760-qubit Advantage 4.1 annealer. To the best of our knowledge, these are the largest factorization problems ever encoded into a quantum annealer. Also, due to the modularity of the encoding, this number will scale up automatically with the growth of the qubit number in future chips. Thus, we believe that this encoding can be used as a baseline for many future research for prime factorization via QA.

Second, we have investigated the problem of actually *solving* encoded PF problems by running an extensive experimental evaluation on a D-Wave Advantage 4.1 quantum annealer. Despite the presence of faulty qubits and couplings and within the limited amount of QPU time we had access to, by exploiting all the encoding and solving techniques we introduced and described in this paper, 8,219,999 = 32,749 $\times$ 251 was the highest prime product we were able to factorize. To the best of our knowledge, this is the largest number which was ever factorized by means of a quantum annealer, and more generally by a quantum device, without adopting hybrid quantum-classical techniques. We are confident that even better results can be obtained with a less-faulty annealer and larger availability of QPU time.

There is still much room for further developments. First, efficient encodings for alternative multiplier schemata could be developed^18^. Second, other solving strategies within the annealing process could be conceived and empirically investigated. Moreover, D-Wave recently announced the upcoming generation of quantum processors built on top of a new topology, Zephyr, that provides more connections and cliques among different sets of qubits. Once we have access to a large-enough Zephyr processor, we plan to test out encoding algorithms to get better penalty functions for the CFAs reach global minima more easily during the solving phase.

### Supplementary Information

Supplementary Information.

## Data Availability

Data about the experimental section, and in particular the code to replicate the solving experiments, is publicly accessible here: https://gitlab.com/jingwen.ding/multiplier-encoder/.
