# Supplementary material for: Effective prime factorization via quantum annealing by modular locally-structured embedding
Source: Sci Rep. 2024 Feb 12;14:3518. doi: 10.1038/s41598-024-53708-7 (PMC10861481; doi:10.1038/s41598-024-53708-7)
Supplement: Supplementary file 1 — Supplementary Information. [file 41598_2024_53708_MOESM1_ESM.pdf]

Effective Prime Factorization via Quantum Annealing by Modular Locally-structured Embedding: Supplementary materials

Jingwen Ding[1], Giuseppe Spallitta[1], and Roberto Sebastiani[*1]

[1]Dept. of Computer Science and Engineering, University of Trento, Trento (Italy)
[*]Corresponding author
[1]{jingwen.ding, giuseppe.spallitta, roberto.sebastiani}@unitn.it

ABSTRACT

Here we provide the CFA Ising models used in the experiments. In particular, we provide both the visual representation of the two CFAs embedded in the Pegasus topology, and the two matrices with the offset, biases and coupling values for both CFAs.

The CFA Ising models used in the experiments

The two Ising models CFA0 and CFA1 from Table 2, which we have used in the experiemnts are presented in Figure S1. The coefficients are represented in the form of matrix Q, so that each value Q_{jj} in the diagonal represents the bias of the jth qubit, and each value Q_{ij} in the upper part above the diagonal represents the coupling between the ith and jth qubit (the matrix is symmetric, so we do not report its lower part below the diagonal). In the matrix the 11 qubits are indexed as follows:
1-4: the horizontal qubits in the main tile, left to right;
5-8: the vertical qubits in the main tile, top to bottom;
9: the leftmost horizontal qubit of the neighbour 120°-degree bottom-right tile;
10-11: the topmost vertical qubits of the neighbour 45°-degree bottom-left tile, top to bottom.

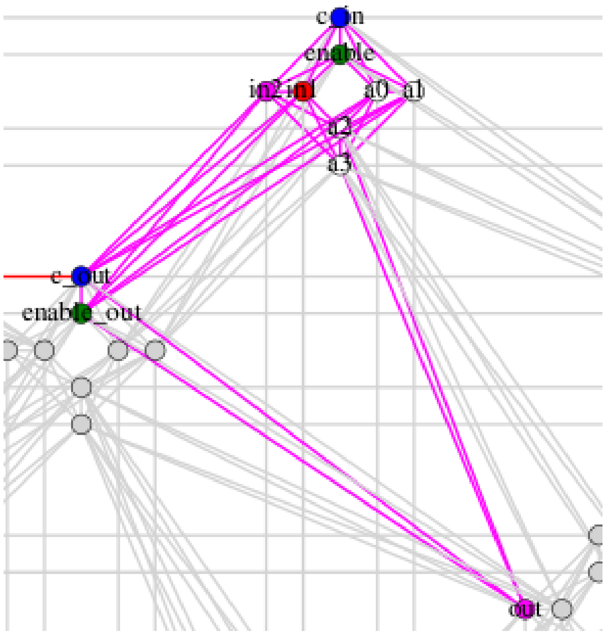

(a) The variable placement for configuration CFA0 of Table 2 .

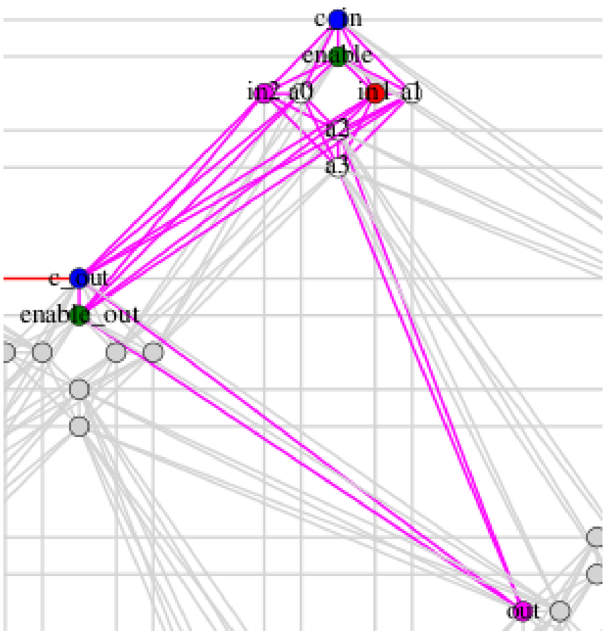

(b) The variable placement for configuration CFA1 of Table 2 .

Q =

	in2	a0	in1	a1	c_in	enable	a2	a3	out	c_out	enable_out
in2	0	1			$\frac{25}{48}$	0	-2	1		-$\frac{73}{48}$	0
a0		0			-$\frac{25}{16}$	0	-2	1		-$\frac{73}{48}$	0
in1			-$\frac{1}{2}$	$\frac{1}{2}$	0	0	1	-1		-1	0
a1				-$\frac{35}{24}$	0	-2	1	-$\frac{71}{48}$		-$\frac{25}{48}$	-2
c_in					0	0					
enable						$\frac{23}{24}$					
a2							-1	-2	-1		
a3								$\frac{25}{48}$	-2		
out									$\frac{23}{48}$	1	$\frac{23}{48}$
c_out										1	-$\frac{23}{48}$
enable_out											0

(c) The coefficients of the Ising model for the configuration CFA1 in Table 2 . ($g_{min} = 2, \theta_0 = \frac{175}{12}$.)

Q =

	in2	in1	a0	a1	c_in	enable	a2	a3	out	c_out	enable_out
in2	-$\frac{25}{96}$	$\frac{1}{2}$			$\frac{1}{48}$	-$\frac{1}{48}$	-$\frac{3}{2}$	1		-$\frac{73}{48}$	$\frac{25}{96}$
in1		-$\frac{1}{2}$			$\frac{1}{2}$	$\frac{25}{48}$	-$\frac{1}{2}$	-$\frac{1}{2}$		-1	-$\frac{1}{48}$
a0			-$\frac{9}{32}$	-$\frac{1}{48}$	$\frac{1}{48}$	-2	-$\frac{23}{24}$	-$\frac{47}{48}$		-$\frac{25}{96}$	-2
a1				-$\frac{65}{96}$	-2	-$\frac{1}{48}$	1	-$\frac{3}{2}$		-$\frac{73}{48}$	$\frac{25}{96}$
c_in					$\frac{7}{16}$	$\frac{1}{48}$					
enable						-$\frac{11}{24}$					
a2							$\frac{47}{48}$	$\frac{1}{2}$	-2		
a3								$\frac{23}{24}$	-2		
out									-$\frac{23}{32}$	1	$\frac{23}{32}$
c_out										$\frac{1}{2}$	-$\frac{23}{96}$
enable_out											$\frac{1}{48}$

(d) The coefficients of the Ising model for the configuration CFA0 in Table 2 . ($g_{min} = 2, \theta_0 = \frac{1367}{96}$.)

Figure S1. The variable placement and the coefficients for the two CFA configurations used in the experiments.
